# Testing normalization process theory in a randomized trial of mental health clinics implementing digital measurement-based care

**DOI:** 10.1186/s13012-026-01485-4

**Published:** 2026-02-09

**Authors:** Nathaniel J. Williams, Mimi Choy-Brown, Nallely Vega, Gregory A. Aarons, Mark G. Ehrhart, Steven C. Marcus

**Affiliations:** 1 Institute for the Study of Behavioral Health and Addiction, Boise State University, 1910 W. University Dr., Boise, ID 83725 USA; 2 School of Social Work, University of Minnesota – Twin Cities, St. Paul, MN USA; 3 The Ballmer Institute for Children’s Behavioral Health, University of Oregon, Portland, OR USA; 4 Department of Psychiatry, University of California, San Diego, CA USA; 5 Department of Psychology, University of Central Florida, Orlando, FL USA; 6 School of Social Policy and Practice, University of Pennsylvania, Philadelphia, PA USA

**Keywords:** Normalization process theory, NPT, Evidence-based practice, Complex health interventions, Implementation mechanisms, Sustainment, Measurement-based care, Mental health, WISDOM trial, Leadership and Organizational Change for Implementation

## Abstract

**Background:**

Normalization process theory (NPT) is one of the most highly cited implementation theories that explains the mechanisms by which new complex health interventions become embedded and sustained in healthcare settings; however, few of its predictions have been subjected to inferential hypothesis testing. In this theory-driven, ancillary analysis of a large hybrid type 3 effectiveness-implementation trial, we tested two NPT predictions: (1) its generative mechanisms of coherence, cognitive participation, collective action, and reflexive monitoring are modifiable in response to deliberate change efforts, and (2) greater enactment of these mechanisms predicts greater future sustainment of complex health interventions.

**Methods:**

The trial tested two strategies to improve the implementation and sustainment of digital measurement-based care in outpatient mental health clinics serving youth. Twenty-one clinics were randomized to either training and technical assistance alone (k = 10) or training and technical assistance plus the Leadership and Organizational Change for Implementation (LOCI) strategy, in which leaders received training, coaching, and consultation to support implementation (k = 11). Six months after implementation strategies concluded, clinicians (*N* = 144) in both arms completed the Normalization MeAsure Development (NoMAD) questionnaire to describe the extent to which NPT mechanisms were enacted in their clinics. The primary outcome was a monthly, clinic-level, binary indicator of measurement-based care sustainment, derived from automatically-generated system usage data, for 16 months after the NoMAD assessment.

**Results:**

The NPT mechanisms were highly responsive to the organizational implementation strategy, which had a large effect overall (NoMAD total score: *d*_*adj*_ = 1.08, [0.63–1.52]) and on individual mechanisms of coherence (*d*_*adj*_ = 1.02, 95% CI = [0.60–1.44]), cognitive participation (*d*_*adj*_ = 1.00, [0.57–1.42]), collective action (*d*_*adj*_ = 0.96, [0.50–1.42]), and reflexive monitoring (*d*_*adj*_ = 1.01, [0.61–1.42]). Greater enactment of NPT mechanisms predicted significantly higher likelihood of measurement-based care sustainment in the month following the NoMAD assessment (adjusted event rate ratio for high versus low mechanism enactment = 2.96, [CI = 1.94–3.99]) and a significantly less steep decline in the log-odds of sustainment over the 16-month follow-up (*b*_*adj*_ = 0.32, *SE* = 0.15, *p* = .032).

**Conclusions:**

The generative mechanisms proposed by NPT are modifiable in response to theoretically-aligned implementation strategies, and greater enactment of these mechanisms predicts greater sustainment of complex health interventions over 16 months.

**Trial registration:**

ClinicalTrials.gov Identifier: NCT04096274 (Working to Implement and Sustain Digital Outcome Measures); Registered September 19, 2019; url: https://www.clinicaltrials.gov/study/NCT04096274

**Supplementary Information:**

The online version contains supplementary material available at 10.1186/s13012-026-01485-4.

Contributions to the literature

- This is the first study to test the predictions of Normalization Process Theory (NPT), a highly cited theory of implementation, using objective, longitudinally collected data within a randomized controlled trial
- Results confirm that the implementation mechanisms described by NPT can be purposefully changed; in this study, NPT’s theorized mechanisms were responsive to a theoretically concordant implementation strategy which improved all four mechanisms to a large degree
- Results also confirmed that sustainment of a newly introduced complex health intervention was more likely during the entirety of a 16-month follow-up period when NPT mechanisms were enacted to a greater degree

## Background

Normalization process theory (NPT) is a mid-range theory of implementation that explains potential mechanisms through which complex health interventions become embedded and sustained in healthcare settings [1, 2]. The theory posits four generative mechanisms: (1) coherence, involving shared sense-making about the intervention’s important characteristics and value, (2) cognitive participation, entailing engagement with the intervention and coming to view it as a legitimate component of work, (3) collective action, including steps to enact the intervention, and (4) reflexive monitoring, encompassing appraisal of ongoing intervention delivery and its effects [1, 3].

Since 2009, NPT has informed hundreds of implementation articles and numerous implementation efforts [4, 5]. These studies have contributed significant depth and breadth of understanding about how individuals across contexts engage in the embedding processes theorized by NPT. However, almost all of this research has used qualitative methods which do not enable inferential tests of deductively generated hypotheses [4, 6]. In a systematic review of 108 NPT-informed process evaluations and feasibility studies [6], 96% used qualitative or mixed methods and none employed experimental designs to quantitatively test NPT’s central predictions.

We argue two NPT predictions should be prioritized for hypothesis testing: (1) its four generative mechanisms (coherence, cognitive participation, collective action, reflexive monitoring) are modifiable through intentional change efforts, and (2) higher levels of mechanism enactment predict greater sustainment of complex health interventions. Testing the first prediction is important because unless the mechanisms described by NPT can be purposefully altered through implementation strategies, the theory only describes what has happened organically and perhaps non-reproducibly in some contexts; it does not offer an effective way to influence implementation. Testing the second prediction is important because if greater enactment of NPT mechanisms does not predict greater sustainment of complex health interventions, the theory is not scientifically valid or practically useful.

We evaluated these hypotheses in a theory-driven, ancillary analysis of data from a large hybrid type 3 effectiveness-implementation trial [7]. The trial tested whether adding a leadership-focused organizational implementation strategy to standard clinician training and technical assistance improved the implementation and sustainment of an evidence-based complex health intervention called digital measurement-based care (MBC) in community mental health clinics. Digital MBC involves session-by-session administration of web-based, patient-reported outcome measures and use of the feedback by clinicians to guide treatment [8–11]. Dozens of randomized controlled trials indicate mental health services are more effective when clinicians use MBC in either digital or non-digital formats [8–10]; however, implementation is often substandard [12–15]. Because digital MBC requires coordinated effort by front-desk staff, clinicians, information technology professionals, clinical supervisors, and organizational leaders [12], organizational factors have been identified as key determinants of implementation success or failure [13–17]. Primary results of the trial showed that the addition of an organizational implementation strategy improved youth-level fidelity to digital MBC by 20 percentage points (23% vs. 3%) relative to training and technical assistance alone [7].

The organizational implementation strategy tested in the trial was Leadership and Organizational Change for Implementation (LOCI) [18]. LOCI provides organizational leaders with training, coaching, and consultation to develop skills and strategies that generate a supportive climate for implementation of a specific health intervention within their setting [7, 19]. First-level leaders, who directly supervise clinicians, receive leadership training and coaching. Both first-level and executive leaders participate in strategic planning sessions to align leadership across levels and develop climate-generating strategies. Although LOCI’s theory of change relies on organizational leadership and climate theories [20–22], its components and hypothesized mechanisms align closely with NPT-informed implementation strategies [23] and NPT’s generative mechanisms [1]. This concordance created an opportunity to test NPT using data from the trial because LOCI could reasonably be expected to activate the NPT mechanisms in practice. Table 1 presents a crosswalk mapping LOCI’s leadership training and climate development components to aligned NPT mechanisms [1], guided by May et al.’s [23] recent description of NPT-informed implementation strategies. In addition, the longitudinal data collected in the trial provided an opportunity to test whether NPT mechanisms, assessed after active implementation efforts had concluded, predicted future digital MBC sustainment.

**Table 1:** Crosswalk of Normalization Process Theory (NPT) generative mechanisms and concordant Leadership and Organizational Change for Implementation (LOCI) strategy components

NPT Mechanism (May & Finch, 2009; [1, 2]	Concordant LOCI Components (Aarons et al., 2015) [18]
**Coherence**	Leadership development plan, addressing dimensions of: ▪ Vision/mission ▪ Communication ▪ Knowledgeable Climate development plan, addressing dimensions of: ▪ Focus
**Cognitive participation**	Leadership development plan, addressing dimensions of: ▪ Proactive ▪ Knowledgeable ▪ Supportive ▪ Perseverant Climate development plan, addressing dimensions of: ▪ Selection for EBP ▪ Recognition ▪ Rewards ▪ Existing Supports ▪ EBP Integration
**Collective action**	Leadership development plan, addressing dimensions of: ▪ Proactive ▪ Supportive ▪ Communication ▪ Available Climate development plan, addressing dimensions of: ▪ Educational supports ▪ Recognition ▪ Rewards ▪ EBP integration
**Reflexive monitoring**	Leadership development plan, addressing dimensions of: ▪ Supportive ▪ Perseverant ▪ Available ▪ Knowledgeable Climate development plan, addressing dimensions of: ▪ Use of data ▪ Existing supports

**Fig. 1:**
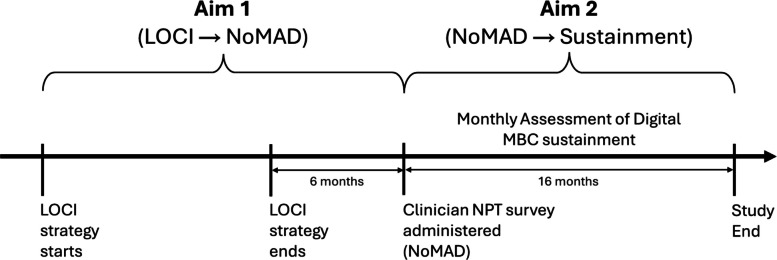
Study aims and timeline for testing Normalization Process Theory (NPT). *Note:* For aims 1 and 2, arrows (→) represent hypothesized relationships between exposures and outcomes. On the study timeline, calendar time is represented by the arrow intersecting all activities. LOCI = Leadership and Organizational Change for Implementation; MBC = measurement-based care; NoMAD = Normalization MeAsure Development questionnaire

In this theory-driven, ancillary analysis of data from the trial, we pursued two aims (see Fig. 1). Aim 1 experimentally tested whether the four NPT mechanisms were responsive to the LOCI strategy. We hypothesized that clinics assigned to LOCI would exhibit superior levels of NPT mechanism enactment assessed six months after LOCI ended, compared to control. Aim 2 tested whether greater enactment of NPT mechanisms predicted superior prospectively-measured sustainment of digital MBC. We hypothesized that clinics with higher levels of NPT mechanism enactment (six months after LOCI ended) would exhibit superior MBC sustainment in the ensuing 16 months.

## Methods

### Setting and design

The trial protocol was prospectively registered at clinicaltrials.gov (Identifier: NCT04096274) and has been described in detail elsewhere [7, 24, 25]. Briefly, within a hybrid type 3 effectiveness-implementation design [26, 27], 21 outpatient mental health clinics that delivered psychotherapy to youths, ages 4–17, in Idaho, Oregon, and Nevada, USA, were randomly assigned to one of two strategies to support digital MBC implementation: (1) training and technical assistance alone (control condition; k = 10), or (2) training and technical assistance plus organizational leader participation in LOCI (LOCI condition; k = 11). Clinics were eligible if they employed 3 + clinicians delivering psychotherapy to youths and were not currently implementing digital MBC. Covariate constrained randomization [28] balanced conditions on clinic size and location. Figure 1 shows the timeline of implementation strategies and assessments for these exploratory aims.

### Measurement-based care

The intervention in this trial was digital MBC, delivered using the web-based Outcomes Questionnaire Analyst (OQ-A) [29, 30]. The OQ-A included youth symptom measures to be completed prior to each treatment session by the youth’s caregiver or, for youths ages 12 +, by the youth and/or caregiver. Feedback from the measures informed clinicians of whether youths were making expected progress in treatment—using a green, yellow, or red alert, showed symptom change over time, and highlighted high risk items endorsed on the most recent measure. The MBC protocol instructed clinicians to administer OQ-A measures at every session and review feedback prior to or within a week of each session. Access to the OQ-A was provided to clinics at no cost for the trial.

### Implementation strategies

Clinic executives and first-level leaders in the LOCI condition began participating in a 12-month version of the LOCI strategy in November 2019. The LOCI strategy provided structured leadership training, individualized coaching calls, and quarterly organizational assessments with tailored feedback reports. Leaders also engaged in guided strategic planning to apply these inputs to their local context. Williams et al. [7] provide details of the LOCI strategy, which was provided to clinics at no cost.

All clinicians and leaders in the trial received an initial one-day MBC training, delivered by the OQ-A purveyor organization in December 2019. Training was followed by two, live, 1-h booster trainings delivered virtually 3 and 5 months later. In addition, all clinics received OQ-A technical assistance from the purveyor organization at no cost from initial training through the trial’s conclusion.

### Data sources and measures

#### Normalization process theory

Clinicians reported on NPT mechanism enactment using the 20-item Normalization MeAsure Development (NoMAD) questionnaire [31]. NoMAD assesses clinicians’ experiences with a focal health intervention, in this case digital MBC, relative to the NPT mechanisms of coherence (4 items; Omega = 0.84), cognitive participation (4 items; Omega = 0.88), collective action (7 items; Omega = 0.86), and reflexive monitoring (5 items; Omega = 0.88). The timeframe for the items is the present (e.g., “Staff in this organization have a shared understanding of the purpose of the OQ-A”) and items are rated on a 5-point Likert scale (*strongly agree* to *strongly disagree*), with one item reverse scored. Scores are calculated for each subscale (mean of items), with higher scores indicating greater enactment, and then combined to yield a total score. Extensive psychometric work supports the reliability, content validity, factorial validity, and measurement invariance of scores on the NoMAD across healthcare contexts [3, 31, 32], including in mental health [33]. The 20 items exhibited strong internal consistency reliability in this sample (Omega coefficient = 0.95).

Data were collected via a web-based survey six months after the LOCI strategy concluded (see Fig. 1). All clinicians working with youths were eligible. Clinic leaders provided the research team with lists of eligible clinicians and their work email addresses. The research team directly emailed clinicians survey invitations. An incentive of $55 USD was provided for survey completion.

#### Sustainment of MBC

Sustainment of digital MBC was assessed monthly using a clinic-level, binary indicator of whether at least one youth had an OQ-A measure administered. Measure administration was logged automatically by the OQ-A system. The threshold of one youth per month was selected because (a) overall usage data suggested any use versus no use provided a reasonable distinction between clinics which had and had not sustained use of MBC, and (b) clinics varied considerably in size such that any choice for minimum number of youths per month would have been arbitrary. The dichotomous monthly indicator of sustainment was logged for each clinic beginning in the month after the NoMAD questionnaire was administered until the study endpoint (16 months total).

### Data analysis

Analyses for aim 1 examined mean differences on clinician-reported NoMAD total score and subscales, contrasting LOCI and control clinics 6 months after LOCI completion using linear mixed models [34] with a random intercept accounting for nesting of clinicians within clinics. Adjusted models included clinic size. Effect sizes were calculated using Cohen *d*, which represents the standardized mean difference between groups [35]. Values of *d* are typically interpreted as small (0.2), medium (0.5), and large (0.8) [36]. Models were implemented in Stata version 17.0 [37] using the mixed command, under maximum likelihood estimation. One extreme clinic outlier was excluded from all analyses based on a clinic-level Cook’s D value 2.6 times higher than the recommended cutoff [38, 39].

Aim 2 analyses examined whether the extent of NPT mechanism enactment in clinics at 6 months post-LOCI predicted the subsequent likelihood and trajectory of MBC sustainment over the following 16 months. For these longitudinal, clinic-level analyses, we used random-intercept logistic regression, incorporating a random intercept for the nesting of observations within clinics, a logit link, and Bernoulli distribution [40] implemented via the xtlogit command in Stata. The model included the standardized clinic-level NoMAD total score, its interaction with time (months since the NoMAD assessment), and covariates of implementation condition and clinic size. Covariates were included to demonstrate the unique association between NoMAD total score and MBC sustainment independent of the effect of implementation condition. To facilitate interpretation, we graphed model-estimated predicted probabilities of sustainment by month for high (75th percentile), medium (50th percentile), and low (25th percentile) values of NoMAD total score, representing different levels of NPT mechanism enactment. We also calculated event rate ratios contrasting the probability of sustainment for clinics with high versus low mechanism enactment in the month after the NoMAD assessment and at the conclusion of the 16-month follow-up period.

## Results

A total of 19 clinics contributed data to the analyses, with one clinic excluded because it closed before NoMAD surveys were administered (see CONSORT flow diagram in Additional File 1). The clinician survey response rate was high (85%) and did not differ significantly between conditions (LOCI = 87%, control = 80%, *p* = 0.201). Clinicians’ NoMAD scores showed substantial clustering at the clinic level (ICC[1] range = 0.31–0.40), suggesting responses were strongly influenced by clinic setting and supporting the use of linear mixed models. Table 2 presents characteristics of the clinics and clinicians included in these analyses.

**Table 2:** Characteristics of study clinics and clinicians

Characteristic	LOCI condition	Control condition	Total
Clinics			
N	11	8	19
State, n (%)			
Idaho	7 (64)	6 (75)	13 (68)
Oregon	3 (27)	2 (25)	5 (26)
Nevada	1 (9)	0 (0)	1 (5)
Legal Status, n (%)			
Non-profit	5 (45)	2 (25)	7 (37)
For-profit	6 (55)	6 (75)	12 (63)
N of youths served (year prior to trial), *M* (SD)	433.1 (298.1)	325.3 (186.2)	387.7 (256.6)
% revenue Medicaid, *M* (SD)	55.6 (26.0)	66.1 (30.1)	60.3 (27.5)
Clinicians			
N	100	44	144
Sex, n (%)			
Male	18 (18)	3 (7)	21 (15)
Female	80 (80)	39 (89)	119 (83)
Prefer not to disclose	2 (2)	2 (5)	3 (2)
Race, n (%)			
Asian	3 (3)	1 (2)	4 (3)
Black or African American	2 (2)	1 (2)	3 (2)
Native Hawaiian or Other Pacific Islander	2 (3)	0 (0)	2 (1)
Prefer not to disclose	5 (5)	2 (5)	7 (5)
Prefer to self-describe	6 (6)	1 (2)	7 (5)
More than one	2 (2)	0 (0)	2 (1)
White	80 (80)	39 (89)	119 (83)
Ethnicity, n (%)			
Hispanic/Latino/a	15 (15)	4 (9)	19 (13)
Years experience in mental health, M (SD)	5.6 (5.5)	5.6 (6.1)	5.6 (5.7)
Years tenure in organization, M (SD)	3.3 (4.1)	2.5 (3.1)	3.1 (3.8)
Age in years, M (SD)	37.8 (10.3)	39.3 (9.6)	38.3 (10.1)
Extent to which education and clinical training addressed MBC (0–4), M (SD)	1.4 (1.0)	1.6 (0.9)	1.5 (1.0)
Level of prior experience with MBC (0–4), M (SD)	1.6 (1.0)	1.7 (1.0)	1.6 (1.0)

Some percentages do not add to 100 due to rounding

*LOCI* Leadership and Organizational Change for Implementation, *MBC* measurement-based care

### Responsiveness of NPT mechanisms to the LOCI strategy

Hypothesis 1 posited that the four NPT mechanisms would be enacted to a greater degree in clinics randomly assigned to LOCI versus control. This hypothesis was supported. Table 3 presents descriptive statistics for the NoMAD total score and subscales by condition, alongside Cohen *d* effect sizes for the adjusted and unadjusted comparisons; all effects were large (*d* range = 0.83–1.08). In both unadjusted and adjusted analyses, clinics randomly assigned to LOCI exhibited significantly higher enactment of coherence (*b*_*adj*_ = 0.85, *SE* = 0.18, *p* = 0.000), cognitive participation (*b*_*adj*_ = 0.92, *SE* = 0.20, *p* = 0.000), collective action (*b*_*adj*_ = 0.77, *SE* = 0.19, *p* = 0.000), reflexive monitoring (*b*_*adj*_ = 0.83, *SE* = 0.17, *p* = 0.000), and overall (NoMAD total score: *b*_*adj*_ = 0.83, *SE* = 0.17, *p* = 0.000).

**Table 3:** Effects of the Leadership and Organizational Change for Implementation (LOCI) strategy on Normalization Process Theory (NPT) generative mechanisms at 6-month follow-up

NPT Mechanism (NoMAD)	*M* (*SD*)	Cohen *d* (95% CI)	
		Unadjusted	Adjusted
Total Score		0.95 (0.46–1.43)	1.08 (0.63–1.52)
LOCI	2.47 (0.67)		
Control	1.73 (0.73)		
Coherence		0.90 (0.44–1.36)	1.02 (0.60–1.44)
LOCI	2.60 (0.77)		
Control	1.86 (0.77)		
Cognitive participation		0.90 (0.45–1.35)	1.00 (0.57–1.42)
LOCI	2.58 (0.83)		
Control	1.74 (0.84)		
Collective action		0.83 (0.33–1.33)	0.96 (0.50–1.42)
LOCI	2.32 (0.73)		
Control	1.64 (0.76)		
Reflexive monitoring		0.87 (0.40–1.34)	1.01 (0.61–1.42)
LOCI	2.47 (0.72)		
Control	1.76 (0.83)		

*N* = 142–144 clinicians nested within *K* = 19 clinics. Cohen *d* expresses the standardized mean difference in the outcome, contrasting LOCI versus control conditions; values generated using linear mixed effects regression. Coherence, cognitive participation, collective action, and reflexive monitoring are subscales combined to produce the NoMAD total score, which reflects all four NPT mechanisms. Adjusted models control for clinic size

*CI* confidence interval, *NoMAD* Normalization MeAsure Development questionnaire

### Association of NPT mechanisms with digital MBC sustainment

**Fig. 2:**
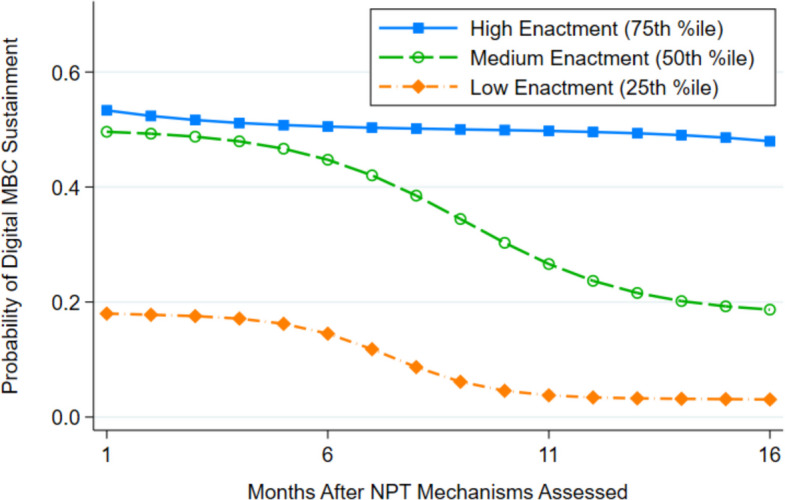
Longitudinal association between extent of Normalization Process Theory (NPT) mechanism enactment and digital measurement-based care (MBC) sustainment. *Note: N* = 304 timepoints nested within *K* = 19 clinics. Markers indicate model-estimated, adjusted predicted probabilities of digital MBC sustainment for clinics at the 25th, 50th, and 75th sample percentiles of NoMAD total scores, reflecting low, medium, and high levels of NPT mechanism enactment, respectively. Analyses are adjusted for implementation condition and clinic size. The event rate ratio in month 1, contrasting the probability of MBC sustainment for clinics with high versus low NPT mechanism enactment was 2.96 (95% CI = 1.94–3.99). In month 16, the event rate ratio contrasting clinics with high versus low NPT mechanism enactment was 15.71 (95% CI = 8.91–22.51). NoMAD = Normalization MeAsure Development (NoMAD) questionnaire

Hypothesis 2 posited that clinics in which NPT mechanisms were enacted to a greater degree six months after LOCI completion would exhibit superior sustainment of MBC in the subsequent 16 months. This hypothesis was supported. Figure 2 shows the association between the adjusted level of NPT mechanism enactment (i.e., NoMAD total score) 6 months after LOCI concluded, and the probability of digital MBC sustainment during the following 16 months, for clinics with high (75th percentile), medium (50th percentile), and low (25th percentile) levels of mechanism enactment. Controlling for clinic size and implementation condition, clinics with higher levels of NPT mechanism enactment had significantly greater log odds of digital MBC sustainment in the first month of the follow-up period (*b*_*adj*_ = 8.81, *SE* = 2.51, *p* = 0.000), resulting in an event rate ratio of 2.96 (95% CI = 1.94–3.99), contrasting the probability of sustainment in clinics with high versus low mechanism enactment. In the 16th month of the follow-up period, the event rate ratio contrasting clinics with high versus low mechanism enactment increased to 15.71 (95% CI = 8.91–22.51). Clinics with higher levels of NPT mechanism enactment also had a significantly less steep decline in log odds of MBC sustainment over the 16-month follow-up period compared to clinics with lesser mechanism enactment (*b*_*adj*_ = 0.32, *SE* = 0.15, *p* = 0.032).

## Discussion

This study is the first to quantitatively test the predictions of an influential implementation theory, NPT—which seeks to explain how newly introduced complex interventions are sustained over time—using longitudinal data from a randomized controlled trial. Results offer robust support for NPT’s predictions. First, the four generative mechanisms hypothesized by NPT to influence complex health intervention sustainment were shown to respond to theoretically-concordant and purposeful change efforts delivered through the LOCI strategy. Within an experimental design, each of the NPT mechanisms and the overall NoMAD total score were significantly higher at 6-month follow-up in clinics exposed to NPT-concordant implementation strategies compared to clinics in a control condition. Moreover, all of the effects were large (*d*s = 0.83–1.08). These findings suggest that NPT not only describes how the implementation of complex health interventions occurs organically in certain contexts, but also identifies mechanisms that can be purposefully targeted and modified [1, 2].

Second, and independently, the generative mechanisms proposed by NPT were shown to predict the future likelihood that a complex health intervention, digital MBC, was sustained for 16 months. This study provides the first quantitative evidence that variation in enactment of NPT mechanisms predicts future sustainment of complex health interventions. While replication in other contexts and with other complex health interventions is needed, these findings suggest users of NPT [41, 42] can expect that greater activation of the mechanisms of coherence, cognitive participation, collective action, and reflexive monitoring will result in improved sustainment of targeted complex health interventions.

This study also suggests that NPT mechanisms may serve as proximal targets or useful surrogate endpoints for assessing sustainment. Because levels of mechanism enactment forecast both the initial probability and the trajectory of sustainment over the subsequent 16 months, future trials could use NoMAD-based thresholds to trigger adaptive implementation supports such as booster leadership coaching in sequential, multiple-assignment randomized designs. Furthermore, because of the substantial difference in trajectories of sustainment shown in Fig. 2, future research may fruitfully identify a cut score on the NoMAD measure beyond which continued sustainment can reasonably be expected and below which continued sustainment is unlikely. For example, in this study, clinics at the 75th percentile maintained a ~ 50% sustainment rate during the entire 16-month follow-up, while clinics with lower NoMAD scores demonstrated clear deterioration.

Like all studies, this research has limitations. Although our measure of sustainment was based on objective system data, future research is needed to examine NPT’s prediction of other sustainment outcomes. Despite the concordance between LOCI’s components and NPT mechanisms, LOCI was not specifically designed to activate NPT mechanisms; consequently, future research that replicates these findings using NPT-focused implementation strategies is needed. In addition, theoretical work is needed to integrate theory on NPT mechanisms, organizational leadership, and organizational climate.

## Conclusions

The generative mechanisms proposed by NPT are responsive to organizationally-focused implementation strategies and greater enactment of these mechanisms predicts greater sustainment of complex health interventions for up to 16 months.

## Supplementary Information

Additional file 1: CONSORT Flow Diagram.

## Data Availability

NJW and SCM had full access to all data in the study and take responsibility for the integrity of the data and accuracy of the analyses. Requests for access to deidentified data can be sent to Dr. Williams at natewilliams@boisestate.edu, Boise State University School of Social Work, 1910 W. University Dr., Boise, ID 83725.

## Abbreviations

LOCI: Leadership and Organizational Change for Implementation strategy
MBC: Measurement-based care
NoMAD: Normalization MeAsure Development (NoMAD) questionnaire
NPT: Normalization process theory
OQ-A: Outcomes Questionnaire-Analyst
USD: United States dollars
