# Supplementary material for: Testing normalization process theory in a randomized trial of mental health clinics implementing digital measurement-based care
Source: Implement Sci. 2026 Feb 9;21:16. doi: 10.1186/s13012-026-01485-4 (PMC12918388; doi:10.1186/s13012-026-01485-4)
Supplement: Supplementary file 1 — Additional file 1: CONSORT Flow Diagram. [file 13012_2026_1485_MOESM1_ESM.docx]

Supplemental Figure 1. CONSORT diagram showing the flow of clinics through the Working to Implement and Sustain Digital Outcome Measures (WISDOM) hybrid type III effectiveness-implementation trial for ancillary NPT analyses.

Clinics contacted to assess interest/ eligibility (k=134)

Randomized (k=21)

Follow-Up

Allocated to LOCI Condition (k=11)

Allocation

Enrollment

Excluded (k=113)

- Did not respond (k=103)
- Did not meet inclusion criteria (k=4)
- Declined to participate (k=6)

Allocated to Control Condition (k=10)

Discontinued study (k=1)

- Clinic closed (k=1)

Discontinued LOCI (k=1)

- Terminated participation (k=1)

Analysis

Analyzed for primary outcomes (k=8)
Excluded from analysis (k=2)

- NPT data not collected due to closure (k=1)
- Extreme outlier (k=1)

Analyzed for primary outcome (k=11)
Excluded from analysis (k=0)

*Note:* Both conditions received training and technical assistance to implement measurement-based care. Control received no additional implementation strategies. Leaders of clinics in the LOCI condition also participated in the 12-month LOCI strategy. LOCI = Leadership and Organizational Change for Implementation; NPT = normalization process theory.
